# Trajectory classification through Freeman’s curve encoding and entropic analysis

**DOI:** 10.1371/journal.pone.0334694

**Published:** 2025-11-04

**Authors:** Roxana Peña-Mendieta, Ania Mesa-Rodríguez, Daniel Estevez-Moya, José Rafael de la Horra, Ernesto Estevez-Rams, Holger Kantz

**Affiliations:** 1 Facultad de Matemática y Computación, Universidad de La Habana, La Habana, Cuba; 2 MPI for the Physics of Complex Systems, Dresden, Germany; 3 Facultad de Física, Universidad de La Habana, La Habana, Cuba; Normandie Universite, FRANCE

## Abstract

The classification of trajectories in two dimensions was done through an entropic analysis of their coded representation. The steps include discretising the trajectory into an 8-symbol code using the Freeman procedure. The resulting sequence is amenable to entropic analysis. Kolmogorov-Sinai entropy, effective complexity measure and informational distance are used. Different classification schemes can be used based on the value of the entropy variables. Two examples are discussed to illustrate the approach: the Hénon-Heiles model, often used as a test bench for complexity analysis and a real experimental case of human posture analysis.

## Introduction

Trajectories in complex systems, in general, can exhibit unpredictable dynamics of stochastic nature mixed with correlations at different scales, allowing some predictability from a given finite portion. The work of Wold [1] shows that time series can be described by a deterministic part that allows predicting the future from the past and an unpredictable part. Discriminating between both components is not trivial and has been approached with different tools in different contexts [2,3].

Trajectories are fundamental in various scientific disciplines and are at the foundation of physics. The essence of a dynamical system lies in examining trajectories, which are solutions to a set of differential equations [4]. Often, while the equations dictating the trajectory are known, their analytical solution is not known or does not exist, and numerical methods are the way to tackle the problem. When this is the case, the researchers are confronted with numerical data from which they must discover patterns and regularities to comprehend the system’s behaviour and predict the trajectory’s course.

In other common cases, the laws governing these trajectories are unknown, and the only available knowledge is the trajectories under different experimental conditions. This, for example, is the case in the study of the mobility of bacteria and other microorganism [5–9]; the wandering of animals and their trajectories [10–12]; the prediction of trajectory of extreme climate events such as hurricanes [13]; studies in human gait [14–19]. In any case, tracking and characterizing trajectories is a challenging task.

A common approach in time series analysis to distinguish between noise and chaos is to build a complexity plot where a magnitude related to patterns or correlations is plotted as a function of some entropy measure [20,21]. Rosso et al. [3] used, to assert structure, the statistical complexity measure introduced in [22], defined in terms of disequilibrium [23] and the probability distribution associated with the time series, as evaluated using the methodology put forward by Bandt and Pompe [24]. The procedure involves a suitable partition of a *D*-dimensional embedding space that exposes the ordinal structure of a given one-dimensional time series. The Bandt and Pompe method is based on the attractor reconstruction, and enough data is assumed to be available for a correct reconstruction. The other sensitive factor is the embedding dimension *D*, and the number of points in the data *N* must comply with the condition N≫D!. This approach has been extended by McCullough et al. [25].

Another relevant approach for time series analysis is the one pioneered by Grassberger et al. [26] and further developed by Crutchfield et al. [27]. It is based on building the minimum computational machine or minimal deterministic automaton, which allows a statistical description of the data [28]. The reconstruction of such a device allows the calculation of pattern-related quantifiers, namely the forecasting complexity or the effective measure complexity, also known as statistical complexity and excess entropy, respectively, and the Kolmogorov-Sinai entropy or entropy density. The use of such a method has yet to go beyond finite state machines [2,26].

In this contribution, a non-parametrized description of a trajectory is proposed, where the signal dependence on time is lacking, and instead, what is known is the actual description of the continuous curve as coordinate tuples (e.g. (*x*, *y*)). We will aim to characterize the balance between unpredictability and structure without using time parametrization in such trajectories. The approach followed has two steps; first, a discretization procedure of the trajectory has to be designed, allowing it to go from a geometric description to a symbolic, finite alphabet description of the trajectories. In the second step, entropic magnitudes will extract information from the coded trajectories, allowing us to reach our goal.

## Method of analysis

### Trajectory encoding

As we will be using Shannon entropy-related magnitudes over a finite alphabet, we need the data as a data sequence over a finite alphabet. Therefore, the trajectory data must be discretized in some convenient way.

When dealing with time series, the character of the data allows the discretization of the signal into a finite number of values that define a finite alphabet. Such partition, assuming it captures the relevant features of interest, once done, makes it possible to estimate the number of relevant magnitudes based on the coded time series [29]. In the case of a trajectory, the scalar character of the signal is, in principle, lost, as the trajectory is usually described in a higher-dimensional space. Discretization, therefore, must follow another procedure.

**Fig 1:**
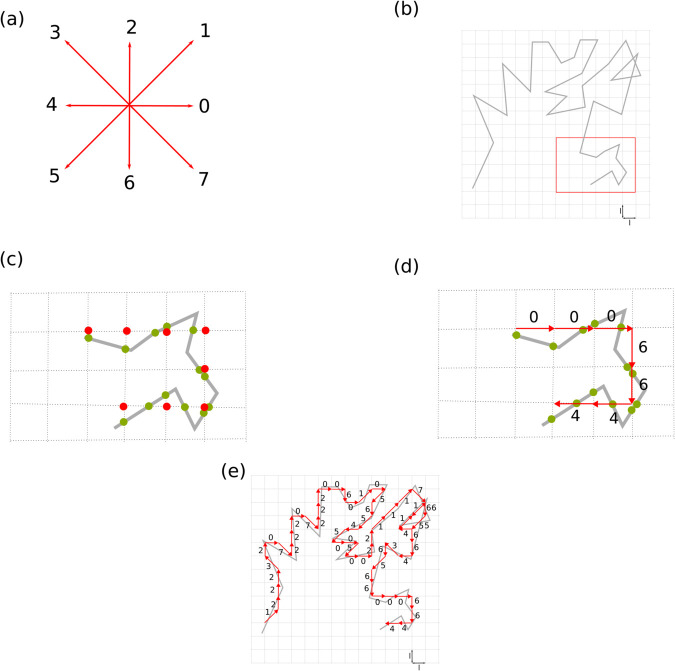
Trajectory encoding. An (a) 8-character alphabet χ is defined for discrete direction, four edges, four diagonals, in the two-dimensional space. Freeman encoding starts with imposing a square grid of length *l* (b) over the trajectory. The intercept of the trajectory with the grid determines the closest corner of the grid (c) to be taken as reference points; (d) from the reference points, the segments are determined, and characters from the alphabet are assigned; (e) the character string of the whole trajectory follows.

Discretizing trajectories is a well-known procedure in computer engineering since the early days of computers. Freeman devised an ingenious procedure for such a task in two dimensions driven by the need to feed data into plotting devices [30]. Such discretization has since been known as Freeman coding. Freeman defined a discrete alphabet of 8 symbols corresponding to eight discrete directions in the two-dimensional space (Fig 1a). The idea, then, is to superimpose a square grid of a given scale over the trajectory (Fig 1b) and determine the intersection of the trajectory to the square edges (Fig 1c). For choosing the scale, the distance between consecutive points in the trajectory is calculated and the smallest, mean and largest value computed, together with the standard deviation. The scale is taken within a standard deviation interval below the mean value. Results are not critical to the choice. From the intersection points, the closest node in the grid is assigned (Fig 1d). In such a way, a discrete sequence describing the trajectory is obtained (Fig 1e), defined over the 8-character alphabet, four edges, and four diagonals [31]. The simple nature of the discretizing algorithm makes it suitable for an efficient implementation [32].

The discretization procedure results in a sequence that is not equidistant in time but instead equidistant in arc length. The accuracy of the Freeman code as a representation of the actual trajectory depends on the grid scale. However, once this is fixed and the code sequence is obtained, all the entropic magnitudes can be used to analyze the trajectory.

### Entropic characterization

Let us first fix some notations. Consider a bi-infinite sequence over an alphabet χ, $f=\ldots f_{i-1}f_{i}f_{i+1}\ldots$, where $f_{i}\in \chi$. A finite subsequence of *f* of length *n* is defined as $f_{i,n}=f_{i}f_{i+1}\ldots f_{i+n-1}$. When used as *f*.,_*n*_ it is meant a particular sequence of length *n*, without regard to its starting position. For a given position *p*, it is understood as the past of *p* the infinite sequence $\overset{\leftarrow }{f}=\ldots f_{p-2}f_{p-1}$ which runs from −∞ to *p*–1. Accordingly, the future refers to the infinite sequence $\vec{f}=f_{p+1}f_{p+2}\ldots$, which runs from *p* + 1 to ∞, sometimes *f*_*p*_ is considered as part of the future for convenience. The partition point *p* is irrelevant in a bi-infinite string of symbols.

The Shannon block entropy can be defined as [33]


$$H_{n}(f)=-\sum_{f_{.,n}}\mathrm{Pr}(f_{.,n})\log\mathrm{Pr}(f_{.,n}),$$

where *P*(*f*.,_*n*_) is the probability of finding a sequence *f*.,_*n*_ as a subsequence *f*_*i*,*n*_, for any *i*, in the sequence *f*. The sum is carried out over all possible sequences *f*.,_*n*_ of length *n* for the given alphabet χ. From the continuity at the limit, 0log0 is taken as 0. The Kolmogorov-Sinai (KS) entropy is then given by

$$h(f)=\lim_{n\to \infty }\frac{H_{n}(f)}{n}=\lim_{n\to \infty }h_{n}(f),$$ (1)

where $h_{n}(f)\equiv H_{n+1}(f)-H_{n}(f)$.

The entropy density (1) is the amount of unpredictability, or randomness, per symbol in the bi-infinite sequence and is equivalent to the entropy density defined in Kolmogorov complexity [33]. A random sequence will have an entropy density of 1 in the base of the alphabet cardinality, or, in the general case, h=log|χ|. For an entirely predictable sequence (e.g. periodic), *h* = 0.

Having *h* to measure randomness, a magnitude related to correlation is needed; this is fulfilled with the effective measure complexity [26], also known as excess entropy [27]. The effective measure complexity is the mutual information between past and present, $E(f)=I[\overset{\leftarrow }{f},\vec{f}]$. The mutual information I(a:b) can, in turn, be given in terms of Shannon entropies I(a:b)=H(a)−H(a|b), and it measures the symmetric informational relation between two processes.

The effective measure complexity can also be taken as [26]

$$E(f)=\sum_{n=0}^{\infty }\left[h_{n}(f)-h(f)\right].$$ (2)

If the past and the future are not correlated, then *E* = 0, if the sequence is periodic with period *P*, E=logP [27]. Plots of *E* vs *h* are known as complexity maps, and they show the balance between randomness and predictability for a system [20].

Finally, a distance measure will be needed to compare sequences. We turn to the information distance that comes from Kolmogorov complexity [34]. Information distance between two sequences d(a:b) is the length of the shortest algorithm that runs in a Universal Turing Machine (UTM) that can compute *a* from *b* [35]. This measure is objective up to a constant value given by the particular UTM. In terms of Kolmogorov complexity

$$d(a,b)=\frac{\max\{K(a|b^{*}),K(b|a^{*})\}}{\max\{K(a),K(b)\}},$$ (3)

*K*(*f*) is the Kolmogorov complexity of the sequence *f* [34]. It complies, up to a constant value, with the symmetry, transitivity and triangular inequality of a distance measure [35].

The above definitions are valid in the limit of a bi-infinite sequence and, in the case of the Kolmogorov complexity, are uncomputable due to the halting problem [33], which leads to the need to estimate the KS-entropy, the effective measure complexity and the informational distance.

For the entropy density, Lempel-Ziv factorization is a common choice (details can be found in [36]). Given the unique Lempel-Ziv factorization of a string, the LZ-complexity *C*_*LZ*_ is the number of factors.

The entropy density is then

$$h=\underset{N\to \infty }{lim sup}\frac{C_{LZ}}{N/\log N},$$ (4)

where *N* is the length of the sequence [37].

The reliability of the LZ estimation of entropy density has been extensively studied and reported (the reader is referred to [38] and reference therein, or [39]) and shown to be optimal in a wide range of practical problems (LZ has been proven to be a good choice even for sequence as short as a few hundreds [38]). For our estimations we use an in-house implementation of the LZ factorization that avoids possible issues with the use of commercial compressors (e.g. gzip) for entropy estimation. An expression for an estimate of the effective measure complexity follows from (2) and (4). A random shuffling algorithm was used as described in [39].

For the estimation of the information distance *d*(*a*, *b*), the following expression is used [40]

$$d(a,b)=\frac{h(ab)-\min\{h(a),h(b)\}}{\max\{h(a),h(b)\}}.$$ (5)

Although not precisely equivalent, this estimation can be related to the one reported in [41]. While [41] uses compressibility of a file in the use of a similar equation as (5), in [40], which is used here, the entropy density is used, estimated by the Lempel-Ziv factorization as described in [36], which differs from that used in compression algorithms. For long enough sequences and high randomness, the finite size of the dictionary in compression software can lead to bias in the estimation of entropy.

### Analysis procedure

**Fig 2:**
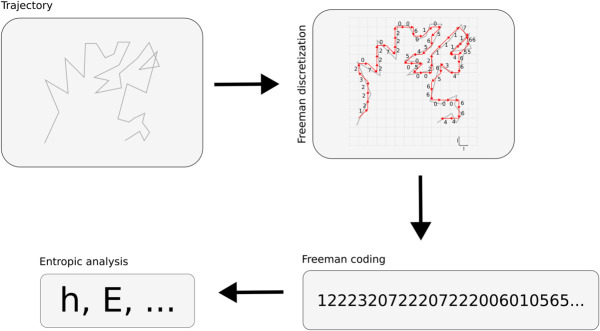
Analysis procedure. For a given trajectory, the Freeman coding is obtained after discretization and treated as a symbolic sequence over which the entropic analysis can be carried out.

Having set how the discretization and coding of the trajectories and the entropic analysis can be carried out, the whole analysis procedure is described in Fig 2.

## Hénon-Heiles trajectories.

**Fig 3:**
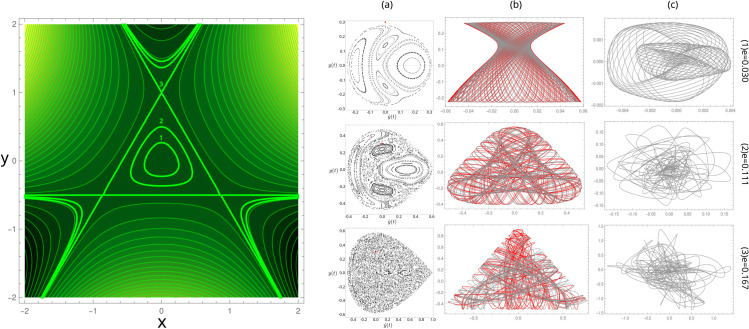
Left: The energy landscape V(x,y) of the Hénon-Heiles potential. Points (1), (2) and (3) correspond to energy isolines of *e* = 0.030, *e* = 0.111 and *e* = 0.167, respectively. **Right:** (a) the Poincaré section at the energy values given at the end of the row; (b) Two orbits, one in black and one in red, with slightly different initial points but the same energy. For increasing energy values, the orbits become more irregular; (c) The difference between the two orbits. As energy increases, the system is more sensible to the initial conditions. Notice that the spatial scales change from one row to the next.

We used the described procedure to analyze the Hénon-Heiles (HH) potential trajectories in two dimensions. The HH potential, originally proposed in the area of astronomical orbits, is a mixed system [42] used as a model for various physical systems of different natures [43]. Mixed must be understood as the coexistence of regular and irregular trajectories. It is defined by [44]

$$V(x,y)=\frac{1}{2}(x^{2}+y^{2})+x^{2}y-\frac{1}{3}y^{3},$$ (6)

which corresponds to two harmonic oscillators with a non-linear coupling, given by the second term in (6) and a non-harmonic factor, affecting one of the oscillators, given by the third term. The potential has a three-fold axis symmetry and is not binding, i.e., there exists a critical energy above which trajectories can escape to infinity (Fig 3, **left**).

The corresponding Hamiltonian equations are

$$\begin{array}{c} \ddot{x}=-x-2xy\\ \ddot{y}=-x^{2}-y+y^{2}. \end{array}$$ (7)

The conservative system exhibits regular and unpredictable orbits as a function of the initial conditions and the total energy [44,45]. The central well has an equilibrium point at the origin (0,0) where the system is nearly integrable and an escape energy of 1/6 at the saddle points (0,1), $\left(-\sqrt{3}/2,-1/2\right)$ and $\left(\sqrt{3}/2,-1/2\right)$ (Fig 3 left). Therefore, for energies above 1/6, at least one escape route exists for long enough trajectories. We will, therefore, be interested in energies below such threshold values where orbits are bounded. According to the Weinstein-Moser theorem, there are guaranteed to exist periodic solutions to the bounded orbits of the HH potential, as has been shown by the calculation of the Poincaré section (Fig 3 right) [44], at low energy, only closed curves are seen covering the whole area. However, at an energy roughly above 1/12, the closed curves in the Poincaré section no longer cover the whole area but instead form a chain of islands between the islands, isolated points corresponding to the same trajectory can be seen distributed at random, showing the occurrence of chaotic orbits. As the energy increases, the number of isolated random points increases. Near the escape energy, they dominate the whole area of the section [44], meaning that for suitable energies, the system displays the coexistence of both regular and chaotic solutions, where the initial condition in phase space determines their nature. In contrast, as a function of energy, one finds the transition from all trajectories being (quasi-) periodic to almost all being chaotic.

**Fig 4:**
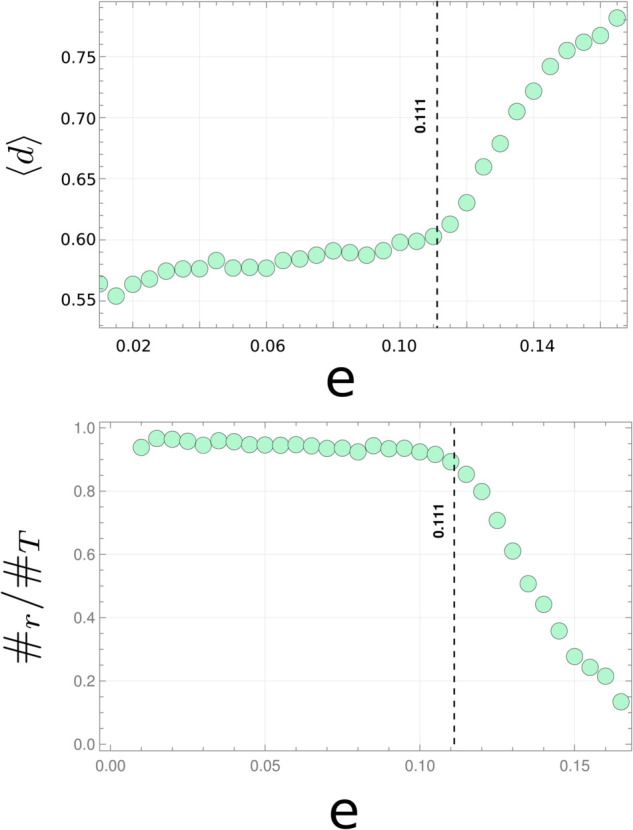
Above: The mean informational distance between two trajectories with close initial conditions as a function of energy e (error bars too small to be represented). **Below:** The ratio between the number of regular orbits ${\#}_{r}$ with respect to the total number of orbits ${\#}_{T}$ as a function of energy. The threshold used to distinguish between regular and chaotic trajectories was taken at 0.6, the value of ⟨d⟩ where the slope changes. The graph shows the ability of the entropic analysis over the chain-coded trajectories to discriminate between regular and chaotic orbits.

The HH model has been used to test procedures that discriminate between regular and chaotic behaviour. Fig 4 shows the result from our analysis of the trajectories using the above procedure. An initial condition and a slightly perturbed one are chosen for fixed energy (The initial position is perturbed in 0.001 while the momentum is adjusted to keep the energy constant). The orbits from both conditions are calculated numerically by solving the corresponding dynamical equation. Each orbit is then chain-coded under the Freeman alphabet, and the informational distance is calculated between the two. This is repeated for 2100 trajectories of length 5000 for each energy, randomly choosing the initial condition under the energy constraint. The mean value of *d* as a function of energy is shown in Fig 4-above. Two regions can be readily identified with different positive slopes. Below an energy value of 1/9, the increase of ⟨d⟩ with increasing energy is slow. This region corresponds to energy values where the dominant type of orbit is regular. Above the energy value of 1/9, the mean informational distance increases rapidly with increasing energy due to the increasing fraction of irregular orbits. When observing the histogram of the informational distance (not shown), we found that the maximum occurring distance shifts towards higher values for increasing energy. In contrast, the spread in the histogram decreases. From the ⟨d⟩ value where the slope changes and the histograms, we fixed a threshold in the informational distance to 0.7, below which orbits are taken to be regular and above which, chaotic. With these criteria, the fraction of regular orbits was calculated as a function of energy and is shown in Fig 4-below. The plot is similar to reported calculations of the same magnitude [44,46].

**Fig 5:**
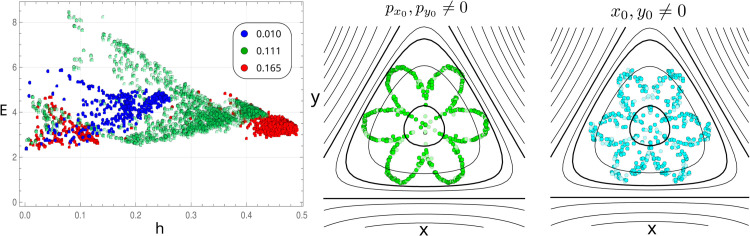
Left: The effective measure complexity-entropy density map, the three colours represent different fixed energies 0.01 (blue), 0.111 (green) and 0.165 (red). For each energy, 2100 orbits were calculated for initial random values. The spread of values resembles an arrowhead. The graph shows that a given entropy density can accommodate a range of structuring given by *E*. **Right:** The entropy density for different directions in space. The value of entropy density is proportional to the distance of the points to the centre. The angular direction in the plot is related to the direction of the perturbed initial condition from the well centre. The energy is fixed at 0.111. The maximum value of *h* is around 0.5. The middle graph corresponds to initial points starting at the well centre (x=0,y=0) but with nonzero initial momentum compatible with the energy choice and pointing in some angular direction. The right graph corresponds to initial points with zero momentum ($p_{x_{0}}=p_{y_{0}}=0$), but out of the well minimum. Again, the angular direction in the plot corresponds to the angular direction of the initial position out of the well centre. In both cases, directions of nearly zero and maximum entropy can be identified. The flower-shaped diagram has a six-fold symmetry, which is compatible with the HH-potential symmetry shown below the plots.

Next, the map of effective complexity vs Shannon-Sinai entropy was calculated and is shown in Fig 5 for three energies: 0.01,0.111,0.165. The energy value determines the *h* region occupied, defining an upper limit value. The corresponding cloud of points forms an arrowhead shape for the two lower energies with two branches, one upper branch going from higher values of effective complexity for low values of entropy density, decreasing as *h* increases. The other branch starts at lower values of *E* and increases with the entropy density. If one considers that the effective measure complexity increases with the length of the patterns in a sequence (for a periodic sequence is equal to the logarithm of the periodicity), then for an energy of 0.01, patterns of higher length are not common, although disorder remains below *h* < 0.25. This can be seen by the values in the upper branch, compared to the same branch when the energy is *e* = 0.111. The smallest *E* values come from the simplest periodic orbits for both energy values, while the higher effective complexity orbits have larger and more complex patterns.

At the border of the escape energy 0.165, mostly all orbits are irregular, as seen by the agglomeration of points at the apex of the plot. However, even for this energy, entropy density is below 0.5, showing that the systems do not accommodate completely random trajectories, and even for the chaotic orbits, predictability remains possible to some extent. Correspondingly, the chaotic trajectories still preserve some structure, as seen by the value of the *E* above 2. From the Weinstein-Morse theorem, it is known that for bounded orbits, periodic solutions always exist, and this is shown in the plot for the *e* = 0.165 as the spear-headed (red) region at the lower side of the *h*. In the case of energies close to the escape value, a gap in the complexity map splits the kind of orbits into two groups: a well-defined chaotic region with *h* values above 0.38 and a small regular orbit region with entropy density below 0.14. What is interesting is the fact that for intermediate energies such as the plotted one *e* = 0.111, trajectories can span a wide range of values in the entropy density. Energy does not fix the possible unpredictability of orbits but merely sets an upper limit. Furthermore, for those intermediate energy values, the effective measure complexity *E* also spans a wide range of values, which goes from 2 to 8, compared to lower or higher energies. This means that such energy values allow a larger number of orbit types in terms of complexity.

The role of the effective complexity measure, as seen in Fig 5, is far from trivial. Complexity-entropy maps allow better knowledge of the balance between disorder and pattern formation. In general, the nature of the plot can be quite different in different systems. One such difference is the range of different pattern formation capacities of a system which can accommodate a given entropy. In a number of systems, this range is pretty narrow for the whole range of entropy values, and indeed, *E* becomes redundant. In the case of the HH system, the reader can observe in Fig 5-left that there is a wide range of pattern-forming capabilities as entropy density decreases for a fixed *h* value, which in turn means that there is a wide range of allowed orbits for a given level of (apparent) disorder. However, Fig 5 also shows this is not a simple relation. There are gaps in the map, pointing out that certain values of *h* allow, with higher probability, a certain amount of correlations than others. If one looks at one energy level, let’s say the one with fixed energy in 0.111 shown in green, one finds that there can be different regions in the plot, showing the non-trivial relation between both magnitudes. This is better seen in the higher energy level points of 0.165, shown in red, where two data point groups are distinguished.

Finally, we fixed the energy at 0.111 and considered two types of initial conditions. In the first case, $x_{0}=y_{0}=0$ and the momentum pointing in all directions of the plane with values compatible with the energy choice; in the second case, the initial momentum was kept to zero $p_{x_{0}}=p_{y_{0}}=0$, and the initial position was taken in all directions compatible with the energy choice. For each initial condition, the entropy density of the corresponding trajectory was calculated after chain-coding. In Fig 5 middle and right, the corresponding graph illustrates the value of the entropy density for each direction as the distance of the points to its centre. Overimposed are the HH potential contours of equal energy. In both cases, when the initial momentum is different from zero in the middle graph and when the initial position is out of the equilibrium point at the right, two non-equivalent (6 in total) directions can be found with entropy density close to zero; and two non-equivalent (6 in total) directions where the entropy density is maximum. The directions of minimum entropy correspond to one escape direction or its opposite (equivalent to the π/4 direction with respect to the horizontal axis), where the energy barrier has the smallest and the steepest increase, respectively. Orbits in both directions are periodic trajectories, moving forward and backward along a line. For the trajectories in the direction with the largest entropy density, the initial momentum makes an angle of 0,π/3 with the horizontal axis. The six-fold symmetry comes from the three-fold symmetry of the underlying potential and an additional π/2 symmetry, which makes equivalent, in terms of entropy density, a given direction and its opposite. The apparent noise character of the nonzero initial position graph (right) is the result of the occurrence of resonant trajectories, a result that will be discussed in further work.

This HH system has been studied before using entropic measure over the time series of each coordinate variable [47]. The idea was to compare the HH behaviour to brain activity, as seen in the electroencephalogram. The authors use Bandt and Pompe methodology [24] as the tool of choice, which needs to fix a time delay and an embedding partition. Permutation entropy is computed as well as the statistical complexity [3,22]. As our purpose is to exemplify our approach, we will not go into much detail, which can be found in [47]. Although correlated variables can be analyzed independently and relevant information of the whole dynamic extracted, using the system actual trajectories seems a more “natural” choice, where the time variable is implicit in the analysis. Entropic analysis over the time series measures each signal against time, and trajectory analysis measures the signals against each other, where time is the underlying variable. Returning to Eq (7), time is implicit, but the Hamiltonian equations can also be seen as correlating both coordinates. In that sense is that we believe that the trajectory analysis, in this particular case, complements previous studies as reported in [47]. A similar comment can be made regarding the approach followed in [48], similar to that of [47].

## Posture analysis

**Fig 6:**
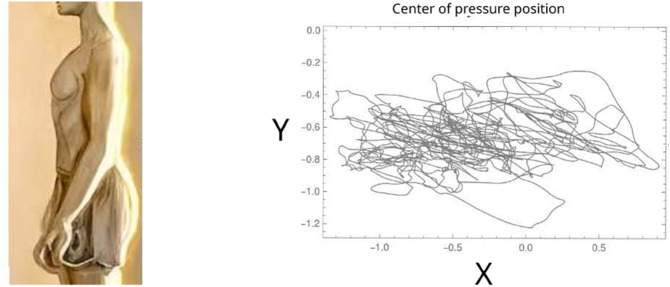
The centre of pressure is measured as a two dimensional trajectory (anterior-posterior and medial-lateral axes) while the subject is standing for 60 seconds over a measuring scale. Four conditions were considered: with eyes closed and open, and standing over a firm surface or foam mat.

This second example uses data from human balance measurement publicly available [17]. One hundred sixty-three participants were asked to stand still for 60 seconds under four conditions: with eyes open or closed, standing on a rigid surface, or on a foam mat. For each condition, three trials were performed. The centre of pressure position in the anterior-posterior and medial-lateral axes as points in two dimensions were recorded while standing still. Details of the experiments can be found in [17]. The change of position of the centre of pressure was taken as a trajectory and coded using the Freeman’s procedure (Fig 6). From the Freeman code, the entropy density, and the effective measure complexity were estimated. The subjects in the experiment were classified according to age and falling events from the previous year of the trials. The analysis is to see if we can discriminate, using the entropic magnitudes, between subjects with no falling events and subjects with falling events.

First, the dataset was divided into four groups according to the conditions of the experiment: eyes open on a firm surface, eyes closed on a firm surface, eyes open on a foam surface, and eyes closed on a foam surface. Similarly, the four datasets were divided into two groups according to age. The balance of the classes was not good, with a higher proportion of patients who had not fallen in the previous year, having around a 75% to 25% ratio in almost all cases. This imbalance can be a problem for the classification task. Therefore, the Synthetic Minority Over-sampling Technique (SMOTE) was applied to balance the classes in the dataset, considering three neighbours per sample to generate the synthetic samples. This technique generates synthetic samples of the minority class by interpolating between the samples of the minority class [49]. This way, the classes are balanced. After applying SMOTE, the ratio was improved to around 56% to 44%.

**Fig 7:**
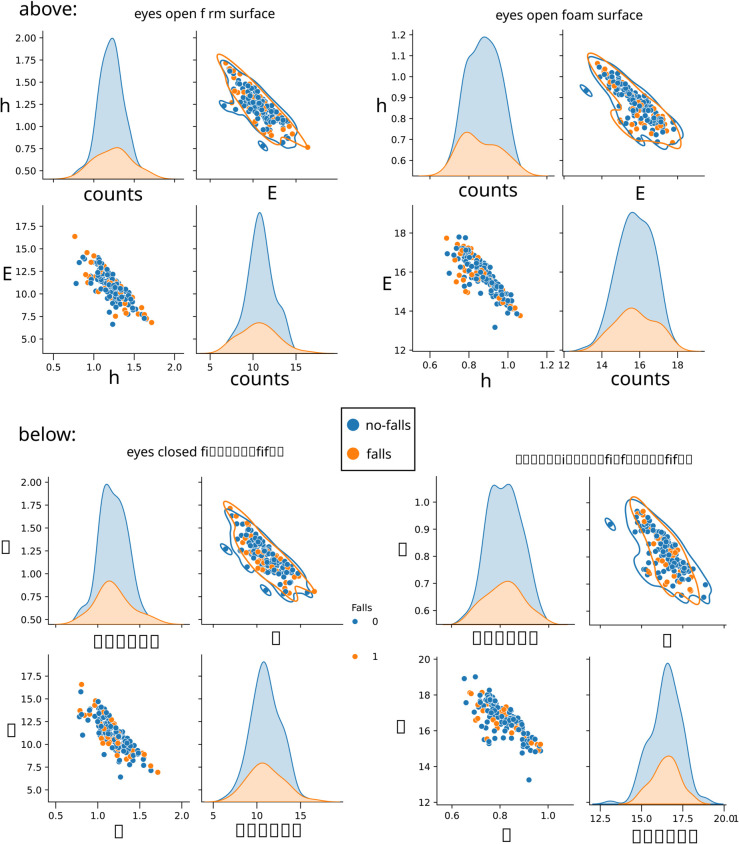
Pairplot visualization of entropy density h and effective measure complexity E for fallers and non-fallers under two representative conditions: above, eyes open on both types of surfaces; below, eyes closed on both types of surfaces. Each off-diagonal subplot shows a scatter plot of one feature against the other (h vs. E), while the diagonal panels display the one-dimensional marginal distributions (histograms) of each feature. The scatter plots reveal heavy overlap between the two classes, with no clear linear separation, and the histograms confirm the similarity of the marginal distributions. These results support the hypothesis that class boundaries are non-linear, motivating the use of neural networks to capture more complex discriminative patterns.

To explore the structure of the data and assess the separability between fallers and non-fallers, a pairplot analysis was conducted, similar in spirit to the complexity-entropy mapping shown in Fig 5. As illustrated in Fig 7, each panel of the pairplot displays scatter projections of entropy density *h* and effective measure complexity *E* in pairwise combinations, while the diagonal panels show the corresponding one-dimensional marginal distributions. Across all experimental conditions, both the scatter plots and the histograms reveal a strong overlap between fallers and non-fallers. No evident linear boundary exists to distinguish fallers from non-fallers in the *h*–*E* space, and the marginal distributions further highlight the subtlety of the class differences. This absence of simple separability suggests that discriminative information is embedded in higher-order, possibly non-linear interactions. For this reason, neural networks were selected as the modeling approach, given their ability to capture such complex relationships and uncover latent patterns that are not easily accessible through traditional linear methods.

Neural networks were used to model the relationship between entropic movement features and fall risk. Given the four experimental conditions—eyes open vs closed and firm vs foam surfaces—and feature combinations per condition, four models were trained per age group. Each model underwent Bayesian optimization using Optuna [50], tuning the number of layers (up to three), neurons per layer (max 128), L2 regularization strength, and learning rate. Five-fold cross-validation was used to select the best hyper-parameters based on mean accuracy. Given the small dataset, regularization was prioritized to reduce overfitting.

Table 1 summarizes the architectures and performance of the trained models for each condition and feature set. The best results in the young group were obtained under the open-firm condition, while for the elderly group, the highest performance was found in the closed-firm condition. This suggests that balance-related entropy features may be more predictive in younger subjects during stable conditions. At the same time, elderly individuals may exhibit more informative patterns in scenarios where balance is challenged. The results, as in the previous experiment, suggest that in elderly groups, other factors influence the motor capabilities of the patients, hence hindering the accuracy of predictions.

**Table 1:** Neural network models architecture and hyper-parameters for each experimental condition.

Group	Condition	Features	Layers	Neurons	Accuracy
Young	Open-Firm	*h*,*E*	2	64, 62	65.22%
	Closed-Firm	*h*,*E*	2	109, 24	63.48%
	Open-Foam	*h*,*E*	2	49, 78	60.00%
	Closed-Foam	*h*,*E*	1	8	57.39%
Elderly	Open-Firm	*h*,*E*	1	118	59.76%
	Closed-Firm	*h*,*E*	3	9, 14, 67	60.00%
	Open-Foam	*h*,*E*	1	41	57.53%
	Closed-Foam	*h*,*E*	2	46, 50	60.58%

Given the relatively modest accuracy levels achieved by individual models across the different conditions—particularly in the elderly group—it became evident that no single configuration consistently outperformed the others. This variability in model performance suggests that each condition may capture complementary aspects of the underlying balance dynamics. To mitigate the limitations of relying on a single model and to enhance predictive reliability, we adopted an ensemble approach that integrates the outputs from multiple models.

An ensemble prediction was made with the trained model, averaging the probabilities of the models trained in each age group and combining predictions from each experimental condition. The final probability was calculated as follows:


$$p_{final}=\frac{p_{1}+p_{2}+\cdots +p_{4}}{4},$$

Where *p*_*i*_ is the probability of falling in the previous year as predicted by each model trained, the final probability was then used to classify the patients. The ensemble criterion was evaluated with the 16 models trained in the young group and the models trained in the elderly group. In the young group, the ensemble criterion improved the classification accuracy with an accuracy of 81%. In contrast, in the elderly group, the improvement was less significant, with an accuracy of 70%. The difference in the performance of the ensemble model between the two groups is consistent with the previous results.

**Fig 8:**
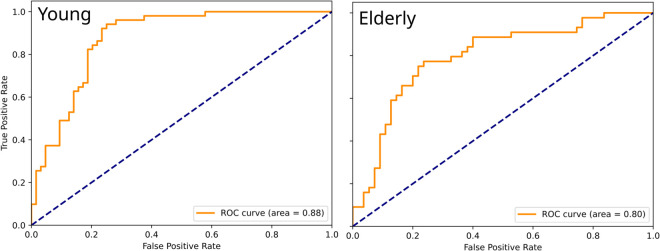
The ROC-AUC curves for the two groups of subject: young and elderly. The plots show the sensitivity (true positive rate) against the unspecificity (false positive rate) as the classification threshold changes. The area value of 0.88 for the young group shows a strong predictive capability, while the elderly group achieves a 0.80 value still indicates a good performance.

To evaluate the overall performance of the ensemble models, the Receiver Operating Characteristic — Area Under the Curve (ROC-AUC) curves were generated for both age groups (Fig 8). The ROC-AUC curve shows the relationship between the true positive rate (sensitivity) and the False Positive Rate (1-specificity) as the classification threshold of a model is adjusted [51]. The ensemble model achieved an AUC of 0.88 for the young group, indicating strong predictive capability. The model effectively balances sensitivity and specificity, making it a reliable classifier for detecting fall risk in younger individuals. The steep initial increase in the true positive rate suggests that the model captures key distinguishing patterns with relatively few false positives.

For the elderly group, the ensemble model performed slightly lower, with an AUC of 0.80. While still a good classifier, the performance drop compared to the young group suggests that fall risk prediction in older individuals is inherently more complex. Factors such as additional comorbidities and broader variability in movement patterns likely contribute to this effect. Nevertheless, the AUC value demonstrates that the model remains valid for classification in this group.

The reported results for young subjects suggest that movement-based entropy features capture meaningful distinctions in this demographic. The elderly group, while still robust, reflects the increased complexity of fall risk assessment due to other contributing factors. Overall, the results indicate that entropy measures hold potential for fall risk classification when combined with neural network models. The ensemble approach improved performance across both age groups, reinforcing the value of integrating multiple experimental conditions.

## Conclusions

To sum up, we have described a procedure for analysing trajectories’ entropy production and pattern formation. The method starts by chain-coding the trajectory using the Freeman code as a choice, but in general, any other discrete coding could be used. The described methodology has been applied to a theoretical model of planetary orbits, allowing us to differentiate between trajectories of different natures, including regular and chaotic. A second example uses real experimental data with an unavoidable mixture of random and predictable components. The entropic magnitudes were used to train a neural network model, which showed a strong predictive capability to classify subjects as healthy ones and those with equilibrium problems that lead to fall events. Both examples are used to exemplify the power of the proposed procedure.

The method is extensible to three-dimensional trajectories, where the Freeman alphabet has a cardinality 26. However, for higher dimensions, the size of the coding alphabet could make it untractable.
